# Multiomics Reveals the Microbiota and Metabolites Associated with Sperm Quality in Rongchang Boars

**DOI:** 10.3390/microorganisms12061077

**Published:** 2024-05-27

**Authors:** Chao A, Bin Zhang, Jie Chai, Zhi Tu, Zhiqiang Yan, Xiaoqian Wu, Minghong Wei, Chuanyi Wu, Tinghuan Zhang, Pingxian Wu, Mingzhou Li, Li Chen

**Affiliations:** 1 Chongqing Academy of Animal Science, Rongchang, Chongqing 402460, China; achao@stu.sicau.edu.cn (C.A.); cq_binzhang@163.com (B.Z.); jiechai91@163.com (J.C.); tuzhi19711@163.com (Z.T.); 18883359161@163.com (Z.Y.); ztinghuan@163.com (T.Z.); wupingxianxian@163.com (P.W.); 2 College of Animal Science and Technology, Sichuan Agricultural University, Chengdu 625041, China; 3 National Center of Technology Innovation for Pigs, Rongchang, Chongqing 402460, China; xiaoqianwu0726@163.com (X.W.); w1883899@163.com (M.W.)

**Keywords:** boar, semen utilization, gut microbiota, fecal metabolites

## Abstract

In this study, we investigated the correlation between the composition and function of the gut microbiota and the semen quality of Rongchang boars. Significant differences in gut microbial composition between boars with high (group H) and low (group L) semen utilization rates were identified through 16S rRNA gene sequencing, with 18 differential microbes observed at the genus level. Boars with lower semen utilization rates exhibited a higher relative abundance of Treponema, suggesting its potential role in reducing semen quality. Conversely, boars with higher semen utilization rates showed increased relative abundances of *Terrisporobacter*, *Turicibacter*, *Stenotrophomonas*, *Clostridium sensu stricto 3*, and *Bifidobacterium*, with *Stenotrophomonas* and *Clostridium sensu stricto 3* showing a significant positive correlation with semen utilization rates. The metabolomic analyses revealed higher levels of gluconolactone, D-ribose, and 4-pyridoxic acid in the H group, with 4 pyridoxic acid and D-ribose showing a significant positive correlation with *Terrisporobacter* and *Clostridium sensu stricto 3*, respectively. In contrast, the L group showed elevated levels of D-erythrose-4-phosphate, which correlated negatively with *Bifidobacterium* and *Clostridium sensu stricto 3*. These differential metabolites were enriched in the pentose phosphate pathway, vitamin B6 metabolism, and antifolate resistance, potentially influencing semen quality. These findings provide new insights into the complex interplay between the gut microbiota and boar reproductive health and may offer important information for the discovery of disease biomarkers and reproductive health management.

## 1. Introduction

Semen quality is the decisive factor affecting the fertility of boars. The most common reason for culling boars is a low semen value (23.7%) [1]. Therefore, the study of the factors affecting the quality of boar semen has important economic value for the swine industry. Semen quality can be influenced by multiple factors, including genetics [2], feed [3], season [4,5], age at semen collection [6], and semen collection interval [7]. Recent studies have suggested that the gut microbe as well as metabolites may influence the male reproductive system [8,9].

The pig intestine is the home for innumerable microbes, most of which are bacteria. The gut flora regulates various physiological processes of the host [10]. The intestinal flora plays regulatory roles through its components and metabolites. Studies have shown that the immune, neurological, endocrine, and reproductive systems are all linked to gut microbes [11,12,13]. More and more studies are beginning to focus on the relationship between gut microbes and the reproductive system. The gut microbiota and its metabolites have been suggested to play a role in the alterations of gonadotropin-releasing hormone (GnRH) [14,15,16], gonadotropins, and sex steroids independently and are therefore likely to contribute to reproductive health. For example, dietary supplementation with Lactobacillus reuteri or other probiotics may be useful in the prevention of male hypogonadism [17], while supplementation with Bacillus paracasei improves sperm density and viability in men with idiopathic oligospermia [18]. These findings support the possibility that the gut microbiota plays a key role in regulating testosterone production and secretion and spermatogenesis. However, the causal relationship between gut microbes and semen quality and its mechanisms remain unexplored [19].

Therefore, in this study, we analyzed semen quality, sperm plasma membrane integrity, microbial composition, and fecal metabolites in Rongchang pig boars with different semen utilization rates. The aim of this study was to reveal the potential link between the gut microbiota and semen quality and to provide a scientific basis for reproductive health management in animal husbandry.

## 2. Materials and Methods

### 2.1. Boars and Sample Collection

Thirty-five healthy Rongchang pig boars aged 31–33 months were selected from Chongqing *Rongchang* Pig National Breeding Farm and were fed with a commercially prepared corn and soybean meal-based diet, as shown in Supplementary Table S1. Semen samples were collected while wearing gloves to maintain sample purity. Sperm suspensions (2 μL) were collected, and the sperm density, overall motility, and forward motility of fresh semen were detected using sperm computer-assisted semen analysis (Minitube, Andro Vision, Tiefenbach, Germany). All these procedures were conducted adhering strictly to contamination-free standards to ensure the accuracy and reliability of the experimental outcomes.

According to the reported methods [20], the semen utilization of each boar was assessed by testing the semen quality for 3 months. Nine boars with <80% semen utilization were randomly selected as the “low semen utilization group” (group L), and nine boars with 100% semen utilization were selected as the “high semen utilization group” (group H). Fresh feces and semen were collected simultaneously and placed in sterile 5 mL cryopreservation tubes. The fecal samples were first placed on ice and then transferred to −80 °C for subsequent analysis.

### 2.2. Measurement of Sperm Plasma Membrane Integrity

The staining of spermatozoa was processed using a LIVE/DEAD Sperm Viability Kit (Thermo Fisher Scientific, Wilmington, DE, USA). Five microliters of diluted SYBR14 dye was added to 1 mL of diluted semen sample to give a final SYBR14 concentration of 100 nM. This was then incubated at 36 °C for 10 min. Then, 5 μL of propidium iodide was added to 1 mL of diluted semen sample to give a final concentration of 12 nM of propidium iodide. The mixture was then incubated for a further 10 min. The samples were then observed under a fluorescence microscope (ZEISS Axio Observer, Oberkochen, Germany) [21].

### 2.3. Boar Feces Microbiota Sequencing

Total genome DNA was extracted from feces using a Magnetic Soil and Stool DNA Kit (TianGen, China, Catalog: DP712). Extracted DNA was quantified using a NanoDrop spectrophotometer (Thermo Fisher Scientific, Wilmington, DE, USA) and diluted to 10 ng/μL with DNase- and RNase-free water. Libraries were constructed according to the published protocols. The V3–V4 hypervariable regions of 16S rRNA genes (341F: CCTAYGGGRBGCASCAG; 806R: GGACTACNNGGGTATCTAAT) were used for PCR amplification. The same volume of 1× loading buffer (containing SYBR green) was mixed with the PCR products and electrophoresis was conducted on a 2% agarose gel for detection. PCR products were mixed in equidensity ratios. Then, the PCR product mixtures were purified using a Universal DNA Purification Kit (TianGen, Shanghai, China, Catalog: DP214). The constructed library was quantified by Qubit and Q PCR (Thermofisher, Santa Clara, CA, USA), and the library was qualified for sequencing using the NovaSeq 6000 PE250 (Illumina, San Diego, CA, USA) platform [22]. For the effective tags obtained previously, denoising was performed with the DADA2 module [23] in QIIME2 software (Version QIIME2-202202) to obtain initial amplicon sequence variants (ASVs) [24]. Species annotation was performed using QIIME2 software. The annotation database used was the Silva aatabase (v.138.1) [25].

In order to analyze the diversity, richness, and uniformity of the communities in the sample, the alpha diversity was calculated from seven indices in QIIME2, including observed_otus, Chao1, Shannon, Simpson, and dominance. The beta diversity was calculated based on weighted and unweighted UniFrac distances in QIIME. Linear discriminant analysis effect size (LEfSe) was conducted to identify bacterial taxa differentially represented between different groups at the genus or higher taxonomic level. PICRUSt2 (V.2.3.0) analysis was used to predict the metagenome functions of the microbiota [26].

### 2.4. LC/MS Non-Targeted Metabolomics Analysis

Metabolites were extracted after sample pretreatment. UHPLC-MS/MS analyses were performed using a Vanquish UHPLC system (ThermoFisher, Langenselbold, Germany) coupled with an Orbitrap Q Exactive TMHF-X mass spectrometer (ThermoFisher, Langenselbold, Germany) at Novogene Co., Ltd. (Beijing, China). Samples were injected onto a Hypersil GOLD column (100 × 2.1 mm, 1.9 μm) using a 12 min linear gradient at a flowrate of 0.2 mL/min. The eluents for the positive polarity mode were eluent A (0.1% formic acid in water) and eluent B (methanol). The eluents for the negative polarity mode were eluent A (5 mM ammonium acetate, pH 9.0) and eluent B (methanol). The solvent gradient was set as follows: 2% B, 1.5 min; 2%–85% B, 3 min; 85%–100% B, 10 min; 100%–2% B, 10.1 min; and 2% B, 12 min. A Q Exactive HF mass spectrometer was operated in positive/negative polarity mode with a spray voltage of 3.5 kV, a capillary temperature of 320 °C, a sheath gas flow rate of 35 psi, an auxiliary gas flow rate of 10 L/min, an S-lens RF level of 60, and an auxiliary gas heater temperature of 350 °C. The raw data files generated by UHPLC-MS/MS were processed using Compound Discoverer 3.3 software to perform peak alignment, peak picking, and quantitation for each metabolite.

### 2.5. Statistical Analysis

All statistical analyses were performed using Student’s *t*-test in SPSS 21.0 software. Spearman correlation analysis between the relative abundance of gut microbiota and plasma metabolites was conducted using GraphPad Prism 8.0. Data were expressed as the mean ± SEM. A *p*-value of <0.05 was considered statistically significant.

## 3. Results

### 3.1. Semen Parameters of Boars

**Figure 1:**
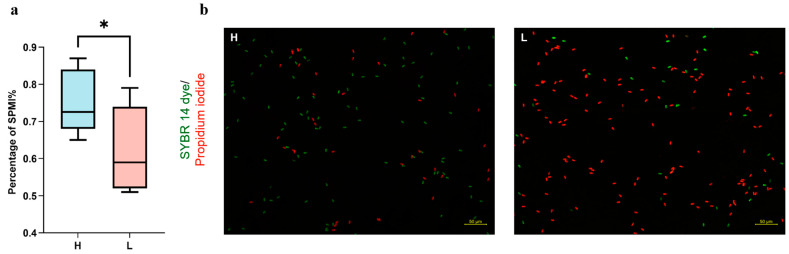
(**a**) Quantitative data for plasma membrane integrity staining. (**b**) Sperm plasma membrane integrity staining. The red color indicates sperm stained with SYBR 14, while the green color indicates sperm stained with propidium iodide. * *p* < 0.05.

Table 1 shows that the difference in sperm density was not significant in the low utilization group compared to the high utilization group, but semen quality parameters such as semen utilization, overall viability, and forward-style viability were significantly higher in group H than in group L (*p* < 0.01). In addition, we stained the collected boar fresh semen with SYBR-14 and propidium iodide, and the results showed that the mean value of plasma membrane integrity was higher in group H than in group L (*p* < 0.05) (Figure 1a,b).

### 3.2. Composition of Gut Microbiota

**Figure 2:**
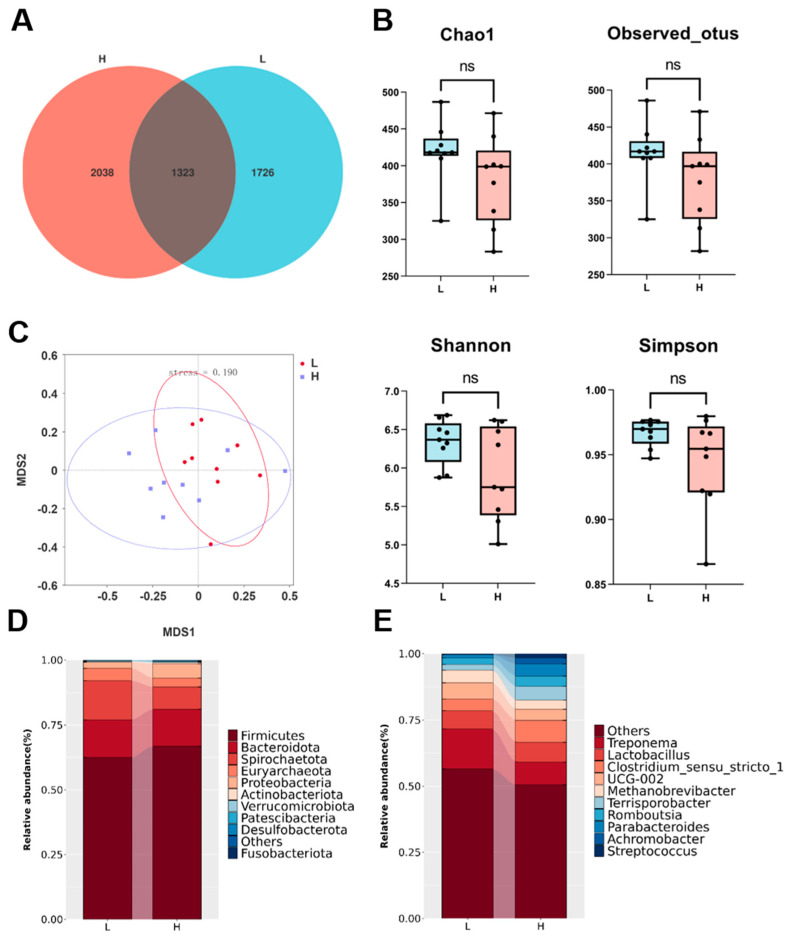
Comparison of the diversity and structure of the gut microbiota between L (low semen utilization) and H (high semen utilization) boars. (**A**) Experimental design and operational taxonomic unit (OTU) distribution between L and H boars. (**B**) Alpha diversity indices of gut microbiota, including observed OTUs, Chao1, Shannon, and Simpson, respectively. ns, not significant. (**C**) Beta diversity presented by non-metric multidimensional scaling (NMDS) analysis based on weighted UniFrac distance. Each data point represents a sample. Linked bar plots of the relative abundances of microbes at the levels of phylum (**D**) and genus (**E**).

To investigate differences in the composition of gut flora between boars with varying semen qualities, we analyzed fecal samples from 18 boars using 16S ribosomal RNA gene sequencing. We obtained 1,401,665 high-quality sequencing reads, with each sample ranging from 64,668 to 86,621 reads. Dilution curves confirmed that the sequencing depth adequately covered the microbial diversity of the samples (Supplementary Figure S1). Subsequently, these reads were clustered into 5087 amplicon sequence variants (ASVs). Specifically, group H exhibited 2038 unique ASV sequences, while group L possessed 1726 unique ASVs. Additionally, 1323 ASVs were shared between the two groups, potentially indicating common characteristics of gut microbiota across both boar groups (Figure 2A). Next, the α and β diversity metrics were calculated to assess the microbial community structures between samples and between groups. The results showed no significant differences in α-diversity (observed ASV, Chao1, Simpson, and Shannon indices) between the two groups of samples (Figure 2B). Non-metric multidimensional scaling analysis (NMDS) based on weighted UniFrac distances showed some variability in fecal flora between the two groups, but with some overlap (Figure 2C).

In both groups of boars, the fecal flora was dominated by *Firmicutes*, *Bacteroidota*, *Spirochaetota*, *Proteobacteria*, *Euryarchaeota*, and *Actinobacteriota*, accounting for more than 95% of the total flora. The relative abundance of *Firmicutes* and *Proteobacteria* in group H was higher than that in group L, and the abundance of *Spirochaetota* in group H was higher than that in group L (Figure 2D). At the genus level, Treponema had a higher relative abundance in group L, while *Parabacteroides* had a higher relative abundance in group H (Figure 2E).

**Figure 3:**
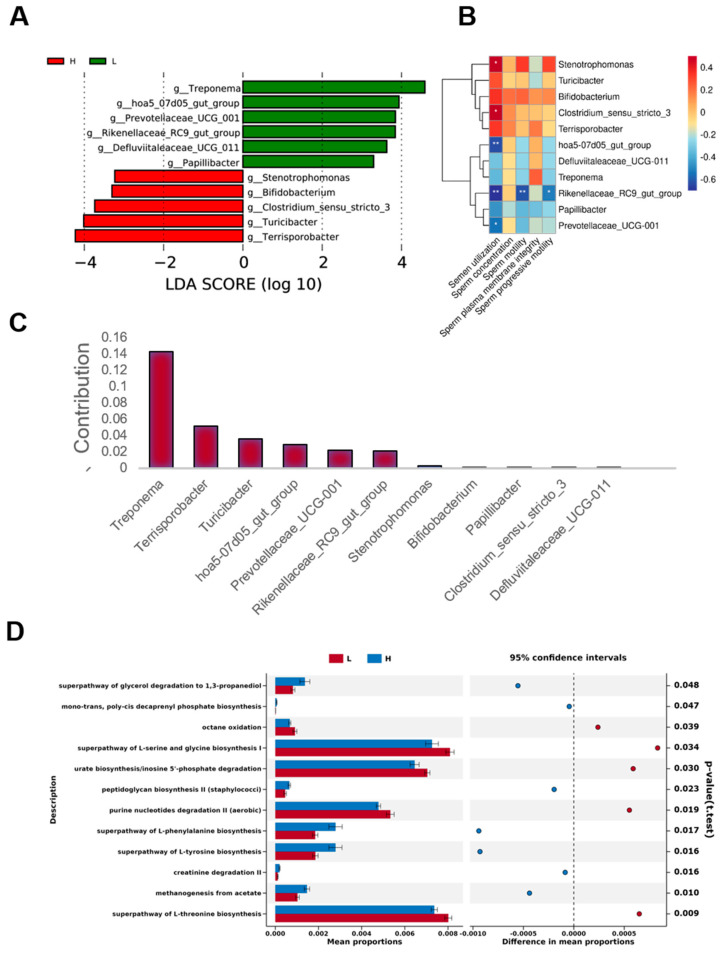
Fecal microbial composition and functional prediction. (**A**) Linear discriminant analysis (LDA) score for discriminated genera in the L and H groups. The LDA score was calculated by LEfSe. The value suggests that it was increased in the two groups (*p* < 0.05, Wilcoxon rank-sum test, LDA > 3.2). (**B**) Heatmap of the Spearman r correlations between the genus-level differences in fecal microbes and the semen parameters of boars. * *p* < 0.05, ** *p* < 0.01. (**C**) SIMPER (similarity percentage) (Warton D I et al., 2012) is a decomposition of the Bray–Curtis difference index that quantifies how much each species contributes to the difference between the two groups. The results show the species contributing to the difference between the two groups. * *p* < 0.05. (**D**) Analyses of pathways predicted by PICRUSt. The Mann–Whitney U test based on the PICRUSt data set revealed differentially enriched bacterial functions associated with either the L group (red) or the H group (blue).

As shown in Figure 3A, we used linear discriminant analysis (LEfSe) to identify fecal microbial markers associated with high and low semen quality. Linear discriminant analysis (LDA) showed that at the genus level, *Treponema*, *YC_ZSS_LKJ147*, *hoa5_07d05_gut_group*, *Rikenellaceae_RC9_gut_group*, and *Prevotellaceae_UCG_001* were the marker microorganisms in the L group, while *Terrisporobacter*, *UCG_001*, *Stenotrophomonas*, and *Clostridium sensu stricto 3* were marker microorganisms in group H (*p* < 0.05, Wilcoxon rank-sum test; log LDA > 2) (Figure 3A). *Stenotrophomonas* and *Clostridium sensu stricto 3* showed a significant positive correlation with semen utilization rates, while *hoa5_07d05_gut_group*, *Rikenellaceae_RC9_gut_group*, and *Prevotellaceae_UCG_001* showed a significant negative correlation with semen utilization rates. *Rikenellaceae_RC9_gut_group* showed a very significant negative correlation with sperm vitality and a significant negative correlation with sperm progressive motility.

SIMPER (similarity percentage) analysis (Warton et al., 2012) is a decomposition of the Bray–Curtis difference index that quantifies how much each species contributes to the difference between the two groups. The results showed that *Treponema*, *Terrisporobacter*, *Turicibacter*, *Clostridium_sensu_stricto_3* contributed the most to the difference between the two groups and their abundance (Figure 3C).

### 3.3. Function Prediction of Gut Microbiota

Based on 16S rRNA sequences, we used PICRUSt analysis to predict the potential functions of the gut microorganisms. By comparing with the KEGG database and examining the functional differences between groups using the t-test method, we found that there were six metabolic pathways with different abundances in the KEGG 3-level pathway classification between boars in group L and group H (Figure 3D). These were categorized and generalized to be related to glycolysis and glucose metabolism, the tricarboxylic acid cycle, choline metabolism, nucleotide metabolism, amino acid metabolism, homo-oligo-fermentation, and the antagonism of naphthoquinone synthesis. The gene expression of guanosine nucleotides degradation III, adenosine nucleotides degradation II, and purine nucleotides degradation II (aerobic) was higher in group L than in group H. The gene expression of super-pathway genes was higher in group L than in group H. In the H group, the expression of genes related to the super-pathway of L-phenylalanine biosynthesis, the super-pathway of L-tyrosine biosynthesis, and the thiazole component of thiamine diphosphate biosynthesis II were significantly higher than in group L. This also extends to the gene content of the pathway of L-tyrosine biosynthesis and the thiazole component of thiamine diphosphate.

### 3.4. Fecal Metabolite Levels in Boars

In order to analyze the differences in metabolites between the intestinal flora of the boars in the low semen utilization group (group L) and the high semen utilization group (group H), we used a non-targeted metabolomics approach to analyze the metabolites qualitatively and quantitatively in the two groups. The results showed that a total of 1015 fecal metabolites were identified by positive and negative ion pattern detection (Supplementary Figure S1), and all metabolites were classified according to the chemical taxonomy annotation of the compounds. Among them, 415 lipids, 196 organic acids and their derivatives, 129 organic heterocyclic compounds, 82 benzenes, 62 organic oxides, 51 nucleosides, nucleotides, and analogs, 44 phenylpropanoids and polyketides, 23 organic nitrogen compounds, and 11 alkaloids and their derivatives were detected.

**Figure 4:**
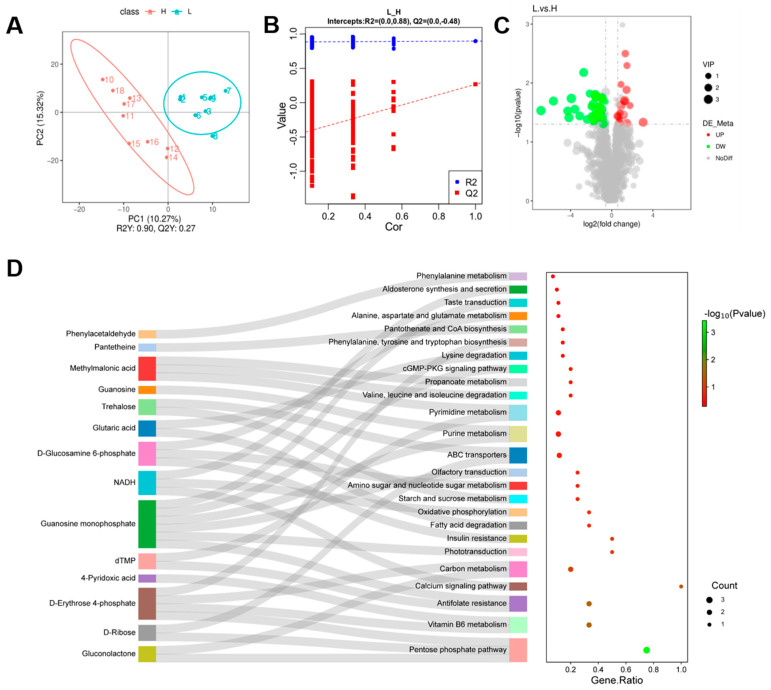
Fecal metabolites. (**A**) PLS-DA score scatter plot. (**B**) Ranking validation diagram. (**C**) Volcano plot of differential metabolites. (**D**) Differential metabolites and the corresponding enriched KEGG pathways.

Partial least squares regression was applied to model the relationship between metabolite expression and sample category to achieve the prediction of sample category. The PLS-DA model was established for each comparison group, and the analysis results are shown in Figure 4A,B, with an R2 value of 0.90, indicating that the model was stable and reliable and not overfitted. Statistical analysis revealed that there were 57 metabolites with statistically significant differences in content between the two groups: 16 metabolites were upregulated and 41 metabolites were downregulated (Figure 4C). These differential fecal metabolites were enriched into the pentose phosphate pathway, vitamin B6 metabolism, pyrimidine metabolism, purine metabolism, carbon metabolism, antifolate resistance, the calcium signaling pathway, and the ABC transporter, as shown in Figure 4D. We performed Spearman’s correlation analysis between differential metabolites and semen indicators, and the results showed that semen utilization was significantly correlated with hydrocinnamic acid, N-(4-chlorophenyl)-N’-(2-phenoxyphenyl) urea, and bicyclo[2.2.2]oct-2-en-1-yl. Meanwhile, 4-methylbenzene-1-sulfonate showed a significant negative correlation with semen utilization. Sperm density was significantly negatively correlated with bicyclo[2.2.2]oct-2-en-1-yl 4-methylbenzene-1-sulfonate.

### 3.5. Microbial and Metabolite Association Analysis

**Figure 5:**
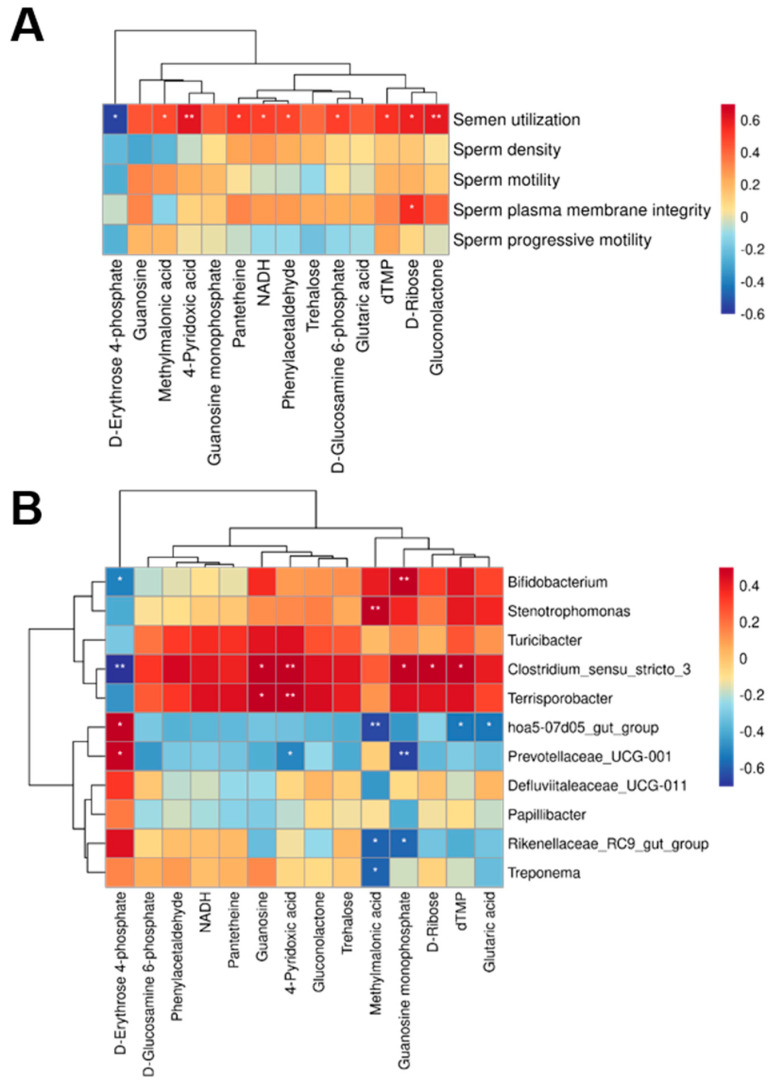
Spearman correlation analysis. (**A**) Correlation between differential metabolites and semen quality. (**B**) Correlation analysis between differential metabolites and differential microbiota. The statistical significance of the results is indicated by * *p* < 0.05, ** *p* < 0.01.

Significantly different genera at the genus level obtained from 16S rDNA analysis and significantly different metabolites obtained from metabolomics analysis were correlated based on Pearson’s correlation coefficient, and heatmaps were drawn to measure the degree of association between species diversity and metabolites in environmental samples. The results regarding differential metabolites and semen utilization showed a significant negative correlation between semen utilization and D erythrose 4-phosphate. Semen utilization was positively correlated with 4-pyridoxic acid, D-glucosamine 6-phosphate, D-ribose, gluconolactone, methylmalonic acid, N4-acetylcytidine, NADH, pantetheine, phenylacetaldehyde, and dTMP. Sperm plasma membrane integrity was significantly and positively correlated with D-ribose (Figure 5A). *Terrisporobacter* was positively correlated with 4-pyridoxic acid and guanosine. *Prevotellaceae_ UCG_001* was significantly negatively correlated with guanosine and 4-pyridoxic acid but positively correlated with D-erythrose 4-phosphate. *Bifidobacterium* showed a significant positive correlation with guanosine monophosphate and a significant negative correlation with D-erythrose 4-phosphate (Figure 5B).

## 4. Discussion

The quality of semen is an important indicator for evaluating the production performance of boars. Changes in the composition of gut microorganisms may affect the level of semen quality. In this study, it was shown that the microbial diversity was lower in the low semen quality group compared to the higher semen quality group. In our study, the gut microbial diversity was significantly different between the two boar groups, but the relative abundance of several genera was significantly different between the two groups at the genus level. In addition, at the phylum level, the fecal flora of both boar groups was dominated by *Firmicutes, Bacteroidota*, *Spirochaetota*, and *Proteobacteria*, which is in agreement with the results of previous studies of the fecal microorganisms of Rongchang pigs and Duroc boars [27]. In our 16S sequencing results, the differential microorganisms we observed were different from those previously reported in York and Duroc boars [28] with different semen qualities, which may have been due to the use of different breeds, feeds, or other factors.

In our study, the abundances of *Terrisporobacter*, *Turicibacter*, *Stenotrophomonas*, *Clostridium sensu stricto3*, and *Bifidobacterium* were significantly higher in group H than in group L. Previous studies have demonstrated that endotoxemia and testicular inflammation are associated with abnormalities in sperm motility and spermatogenesis [29] and that the abnormal proliferation of intestinal microorganisms may increase the permeability of the intestinal mucosal barrier, which can lead to systemic inflammation and subsequent testicular inflammation to reduce semen quality. *Bifidobacterium* in the gut can promote the balance of beneficial bacteria [30]. Probiotics can promote intestinal growth and strengthen the barrier function of intestinal epithelial cells in mice with colitis, thus reducing the degree of colonic damage and maintaining the stability of intestinal microecology [31]. This also helps to reduce the multiplication of harmful microorganisms in the intestine and lower the level of potential inflammatory factors [32], thus achieving an improvement in the overall physiological condition of the boar and positively affecting semen quality. *Bifidobacterium* has been shown to play a key role in A10-FMT to improve the gut microbiota dysbiosis induced by the stimulation of butylaminobenzene sulfonate [33]. A correlation analysis of colonic inflammatory factors and metabolite short-chain fatty acids showed that acetic acid levels showed a significant positive correlation with Treg and IL-10 and that acetic acid in the intestine was able to reduce intestinal inflammation by upregulating the levels of IL-10 and Treg cells. Members of the genus *Terrisporobacter* are strictly anaerobic fermenters, and acetic acid is the main end product of their metabolism [34]. In addition, Zhang et al. showed that the abundance of *Terrisporobacter* was higher in the cecum of healthy piglets than in that of weak piglets and was closely related to piglet health in their study. This suggests that *Terrisporobacter* spp., which were enriched in our group H, may reduce inflammation through the metabolite acetic acid [35]. The genus *Turicibacter* is an important member of the mammalian gut microbiota. Studies have shown that *Turicibacter* strains contain a range of bile salt hydrolases that promote the dissociation of bile salts in the gut to increase the level of unbound primary bile acids in the host serum [36], and studies have demonstrated that a decrease in bile acid levels leads to abnormalities in the metabolism of vitamin A, which can then be passed through the bloodstream affecting the testes, thereby damaging spermatogenesis. We hypothesize that these beneficial microorganisms influence the physiological status of the organism by inhibiting the growth of harmful microorganisms, producing acetic acid and promoting the absorption of bile acids in the intestinal tract, thus influencing the regulation of semen quality.

*Treponema*, *hoa5_07d05_gut_group*, *Rikenellaceae_RC9_gut_group*, *Prevotelaceae_UCG_001*, *Defluviitaleaceae_UCG_011*, and *Papillibacter* were more abundant in group L than in group H. *Treponema* is a common pathogenic microorganism in the intestinal tract of pigs; it is commonly found in piglets during the weaning period due to immunocompromise, which often leads to an increase in the relative abundance of dense spirochetes [37,38,39]. It has been suggested that the increase in spirochetes may be due to decreased immunity.

The fecal metabolome largely reflects the composition of the intestinal flora [40]; therefore, we explored the intestinal metabolites of boars with high and low semen utilization by means of a non-targeted metabolome. We found significant differences between the fecal metabolites of the two groups. Differential metabolites were enriched in the pentose phosphate pathway, vitamin B6 metabolism, and antifolate resistance.

Vitamin B6 is recognized as an indispensable micronutrient universally. Its involvement as a cofactor in the generation of metabolites with immunomodulatory functions is well established [41]. Intestinal microbiota can endogenously synthesize vitamin B6. Epidemiological studies have found associations between consuming foods rich in vitamin B6 and better mental health [42]. The biosynthesis of adrenaline, dopamine, and serotonin all require the involvement of vitamin B6. Additionally, research suggests that supplementing with vitamin B6 leads to elevated levels of 5-hydroxytryptamine (5-HT) in the brain [43]. 5-HT is a monoamine neurotransmitter, primarily found in the gastrointestinal tract, and together with dopamine, it also influences libido. Semen from boars with higher libido levels often exhibits better sperm vitality and motility. Within this context, D-erythrose 4-phosphate, an intermediate metabolite in prokaryotic vitamin B6 synthesis [44], exhibited a marked elevation in the fecal metabolites of the lower semen utilization group (L) compared to the higher utilization group (H). Furthermore, D-erythrose 4-phosphate, a precursor to glyceraldehyde-3-phosphate and fructose-6-phosphate in the pentose phosphate pathway, has been identified as a characteristic intermediate metabolite of Brucella abortus, known for inducing severe reproductive organ damage in animals [45].

D-ribose, a naturally occurring component that is integral to cellular energy metabolism, demonstrated higher concentrations in group H relative to group L. The supplementation of exogenous ribose was observed to alleviate the rate-limiting step of glucose-6-phosphate dehydrogenase (G6PDH) in the pentose phosphate pathway in previous studies, subsequently elevating the level of phosphoribosyl pyrophosphate (PRPP) [46,47]. This acceleration in purine nucleotide synthesis within cardiac and skeletal muscles, facilitated by increased D-ribose levels [48], suggests potential implications for the restoration of the ATP pool. Additionally, the heightened D-ribose levels were posited to directly enhance PRPP levels in sperm cells, potentially influencing purine nucleotide synthesis and ATP pool restoration. Furthermore, D ribose exhibited a plausible role in balancing intestinal flora by suppressing the proliferation of specific deleterious microorganisms.

Gluconolactone, an integral component of the pentose phosphate pathway, furnishes phosphoric ribose for nucleic acid biosynthesis and supplies NADPH, and it has emerged as a pertinent focus. Its endowed attributes, including metal chelation, moisturization, and antioxidant activities, have been delineated [49]. The capacity of gluconolactone to scavenge free radicals and elevate the NADPH/NADP ratio in cardiomyocytes has been highlighted, suggesting the potential augmentation of its cellular reactive oxygen species-scavenging capabilities [50].

Additionally, we observed that dTMP and guanosine monophosphate (GMP) were co-enriched in the antifolate resistance pathway. As a purine nucleotide, GMP is not only an important component of RNA but can also be converted into its deoxyribose form (dGMP) during DNA synthesis [51]. On the other hand, dTMP is an indispensable part of DNA synthesis and repair, directly participating in the construction of the DNA strand and providing the crucial thymine base for DNA [52]. Interestingly, in our study, GMP showed a positive correlation with the beneficial bacteria genus *Bifidobacterium*, suggesting that GMP may directly promote microbial growth or enhance their metabolic activity. Nucleotides and their derivatives can sometimes act as antioxidants, protecting cells from oxidative stress damage. This finding implies that the gut microbiota may modulate folate utilization by influencing the activity of key enzymes involved in folate metabolism, consequently impacting DNA synthesis and spermatogenesis. Furthermore, akin to gluconolactone’s role in nucleic acid biosynthesis, an unfavorable environment with exposure to harmful chemicals such as pesticides [53], heavy metals [54,55], and other pollutants [56] can directly compromise sperm DNA integrity. Hence, fostering a favorable living environment is pivotal to preserving optimal reproductive health in boars.

Both our study and previous research [20,57] have demonstrated that the interaction between gut microbiota composition and fecal metabolites is one of the factors influencing boar semen quality. Specifically, the abundance of beneficial microorganisms such as *Terrisporobacter*, *Turicibacter*, *Stenotrophomonas*, *Clostridium sensu stricto3*, and *Bifidobacterium* may positively influence semen quality by promoting intestinal health and reducing inflammation. Conversely, the enrichment of pathogenic microorganisms like *Treponema* in the lower semen utilization group may contribute to intestinal dysbiosis and compromised reproductive function. Furthermore, alterations in fecal metabolites related to the pentose phosphate pathway, vitamin B6 metabolism, and antifolate resistance suggest potential pathways through which gut microbiota and metabolites modulate semen quality. Specifically, the pentose phosphate pathway is a crucial pathway for intracellular glucose metabolism [58,59], closely associated with the biosynthesis of nucleotides, which are the building blocks of DNA and RNA, essential for maintaining the integrity and stability of sperm DNA. Vitamin B6 plays a significant role in immune regulation and neurotransmission [43], potentially indirectly impacting semen quality by influencing sperm DNA synthesis and repair processes. Changes in the antifolate resistance pathway may affect folate metabolism and utilization, thereby influencing DNA synthesis and spermatogenesis. Beneficial microorganisms may improve semen quality by promoting the production of metabolites involved in pathways such as the pentose phosphate pathway, vitamin B6 metabolism, and antifolate resistance. Conversely, harmful microorganisms may lower semen quality by inhibiting these metabolic pathways. Therefore, the modulation of these metabolic pathways may be one of the key mechanisms through which gut microbiota and metabolites influence semen quality.

## 5. Conclusions

This study is the first to reveal the correlation between semen quality and gut microbiota in Rongchang pigs. We found that semen utilization rates are associated with specific microbes and metabolites. For example, *Treponema* is more abundant in boars with lower semen utilization rates, while *Terrisporobacter* are more abundant in boars with higher semen utilization rates. In addition, we found that certain metabolites vary in concentration between groups with different semen utilization rates. These findings offer a fresh perspective on how gut microbiota influence semen quality, suggesting a potential link between microbiota, metabolites, and semen quality. However, it is essential to acknowledge the study’s limitations as a preliminary investigation. The functional validation of identified key microorganisms and metabolites was lacking. Future research will aim to identify pivotal bacterial strains associated with semen quality, conduct targeted metabolic studies, and validate key metabolites functionally.

## Figures and Tables

**Table 1:** Semen quality parameters of Rongchang boars in different semen utilization rate groups.

Semen Quality Parameters	H	L	*p*-Value
Semen utilization, %	96.86 ± 6.54	61.19 ± 18.94	0.002
Sperm motility, %	83 ± 2.61	69.26 ± 9.31	0.002
Sperm density, 10⁶/mL	203.96 ± 116.62	194.27 ± 67.47	0.832
Sperm progressive motility, %	71.75 ± 2.79	55.32 ± 11.82	0.003

## Data Availability

The study data are present in the main text, and for further inquiries, please contact the corresponding author.

## Supplementary Materials

The following supporting information can be downloaded at: https://www.mdpi.com/article/10.3390/microorganisms12061077/s1, Figure S1: Composition of fecal; Figure S2: Alpha diversity rarefaction curve; Table S1: Composition and nutrient analysis of basal diet.
