# Supplementary material for: Multiomics Reveals the Microbiota and Metabolites Associated with Sperm Quality in Rongchang Boars
Source: Microorganisms. 2024 May 27;12(6):1077. doi: 10.3390/microorganisms12061077 (PMC11205614; doi:10.3390/microorganisms12061077)
Supplement: Supplementary file 1 [file microorganisms-12-01077-s001.zip › microorganisms-2929955-Supplementary Materials.pdf]

Supplementary Materials:

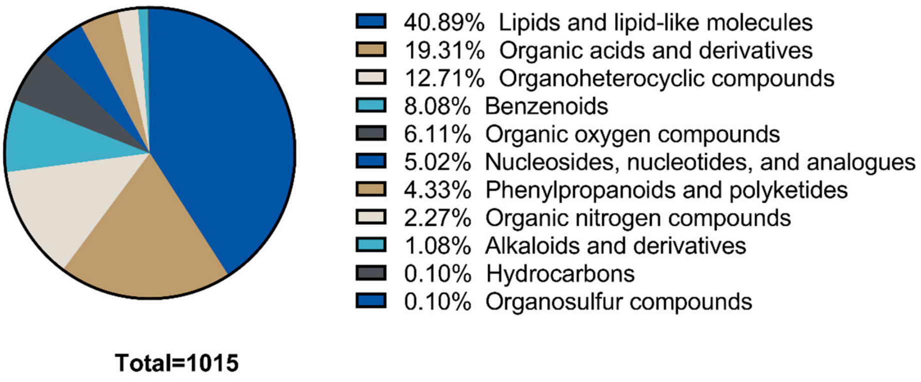

Supplementary Figure S1. Composition of fecal metabolites.

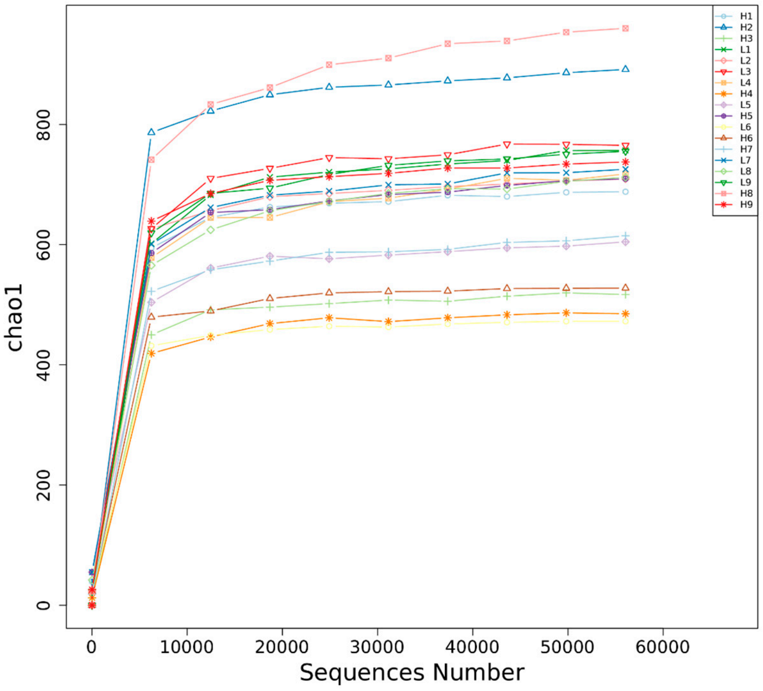

Supplementary Figure S2. Alpha diversity rarefaction curve.

Supplementary Table S1. Composition and nutrient analysis of basal diet.

Ingredient	Content, %
Corn	60
Rice Bran Meal	15
Soybean Meal	12
Extruded Soybean Meal	5
Glucose	2
Imported Fishmeal (Super)	2
Calcium Carbonate	1.5
Dicalcium Phosphate	0.9
Sodium Chloride	0.5
L-Lysine	0.3
DL-Methionine	0.1
Threonine	0.1
Premix	0.6
**Nutritent, %**	
Crude Protein	15.50
Calcium	1.00
Total Phosphorus	0.74
Lysine	0.90
Methionine	0.35
Threonine	0.66
Crude Fiber	2.90
Crude Fat	3.70
Crude Ash	6.40
